# Membrane-Associated Proteins in *Giardia lamblia*

**DOI:** 10.3390/genes9080404

**Published:** 2018-08-10

**Authors:** María C. Touz, Constanza Feliziani, Andrea S. Rópolo

**Affiliations:** Instituto de Investigación Médica Mercedes y Martín Ferreyra—INIMEC-CONICET—Universidad Nacional de Córdoba, Friuli 2434, Córdoba 5000, Argentina; cfeliziani@immf.uncor.edu

**Keywords:** PX domain, variant-specific surface protein, palmitoylation, phosphoinositides, lysosomal vacuoles

## Abstract

The manner in which membrane-associated proteins interact with the membrane defines their subcellular fate and function. This interaction relies on the characteristics of the proteins, their journey after synthesis, and their interaction with other proteins or enzymes. Understanding these properties may help to define the function of a protein and also the role of an organelle. In the case of microorganisms like protozoa parasites, it may help to understand singular features that will eventually lead to the design of parasite-specific drugs. The protozoa parasite *Giardia lamblia* is an example of a widespread parasite that has been infecting humans and animals from ancestral times, adjusting itself to the changes of the environment inside and outside the host. Several membrane-associated proteins have been posted in the genome database GiardiaDB, although only a few of them have been characterized. This review discusses the data regarding membrane-associated proteins in relationship with lipids and specific organelles and their implication in the discovery of anti-giardial therapies.

## 1. Introduction

The genus *Giardia* constitutes a group of unicellular eukaryotic organisms within the large group Excavata, which is characterized by its morphological simplicity [1]. It has been hypothesized that the absence of complexity in this group is a consequence of two fundamental conditions: the early separation of the eukaryotic trunk and the adaptation to parasitism through a process of reductive evolution [2,3]. Still, the evolutionary mechanisms behind the cellular characteristics of these organisms are unknown. 

*Giardia lamblia* (syn. *Giardia intestinalis*, *Giardia duodenalis*) is a unicellular eukaryotic parasite that has attracted scientific attention not only for its worldwide dissemination but also for its particular endomembrane system. The vegetative form of the parasite, the trophozoite, contains two nuclei surrounded by their respective nuclear membranes (NMs), which are in close contact with the endoplasmic reticulum (ER) that covers bilaterally almost the whole body of the parasite [4]. It also contains mitosomes, highly adapted forms of mitochondria, which are surrounded by two membranes, do not contain DNA, and do not produce ATP, but participate in the formation of iron–sulfur clusters [5]. 

Besides the NMs, the ER, and the mitosomes, *G. lamblia* possesses peripheral vacuoles (PVs), which are located below the plasma membrane (PM) of the trophozoites and function as both endosomes and lysosomes [6]. The maintenance of the homeostasis of these organelles during their life cycle is crucial for the survival of the parasite and implies a series of coordinated events in which protein trafficking is central. 

Even with an endomembrane system reduced to the NMs, ER, and PVs, the selective sorting and trafficking of membrane proteins takes place in this organism, although with some particularities. Although the membranes of intracellular organelles are formed by the semipermeable barrier of the lipid bilayer, the properties of a particular membrane cannot be attributed only to the lipid composition. Rather, the type of proteins associated with the lipid layer will delineate the function of a biological membrane. In most eukaryotic organisms, non-cytosolic proteins and lipids are synthesized in the ER and reach the target organelle once they have been selected and directed from the Golgi apparatus. In *G. lamblia*, instead, proteins with a signal peptide are directly sorted from ER exit sites to the PVs or to the PM, while lipids are rarely synthesized de novo but are obtained from the small intestine environment [7,8]. Moreover, an unusual de novo synthesis of secretory vesicles occurs only during the differentiation of the *G. lamblia* trophozoite into cyst, which involves a series of protein synthesis and membrane arrangement events [7]. 

How proteins and lipids are transported, recycled, and modified is still a matter of study, but it is clear that advances in this field will help us to understand the similarities as well as the differences between *G. lamblia* and its host and aid us in the discovery of new specific targeted drugs. This review discusses the data regarding membrane-associated proteins that have been experimentally analyzed, in relationship with lipids and specific organelles and their implication in the discovery of anti-giardial therapies. 

## 2. Fat Attachment: Phosphoinositide-Binding Proteins

The analysis of trophozoite membranes showed that they contain at least two species of phosphatidylinositol (PI) [9], but there is no published data so far regarding the direct presence of phosphoinositides (PIPs), which are the temporal and spatial regulators of membrane trafficking and cell signalling [10,11]. Nevertheless, the expression of two PI3-kinases and one PI4-kinase has been reported, supporting the existence of modified phosphatidylinositol [12,13]. Besides, several proteins containing PIP-binding domains have been characterized in *G. lamblia*, favoring the existence of PIPs and suggesting that there may be a combination of organelle–membrane selectivity and protein function. 

The FYVE (Fab1p, YOTB, Vac1p, and EEA1) domain: In 2010, Sinha et al. reported a protein (GL50803_16653) containing a conserved FYVE domain, which is expressed during the cell cycle of *G. lamblia* [14]. It has been reported that proteins containing the FYVE domain are able to bind to PI3P located in the endosomal and lysosomal compartments in mammalian and yeast cells [15], and analysis of lipid binding using the Protein Lipid Overlay (PLO) assay showed that the *G. lamblia* FYVE domain alone possesses higher binding affinity for PI3P than for PIs and PI4P [14]. The expression of the fusion of green fluorescent protein (GFP) and the giardial FYVE domain in yeast showed that it localizes to endosomes [14]. Considering that PI3P is present in endosomes, PI4P is in the Golgi, and PIs are distributed in the ER and vacuoles in yeast cells, it is possible that GL50803_16653 interacts in *G. lamblia* with the PV membranes, the all-in-one endosomal–lysosomal compartment in this parasite. In line with this, since the FYVE domain is present in Vps27, an ESCRT-0 complex subunit that initiates the multivesicular body (MVB) sorting pathway at the endosomal membranes in other cells via its binding to PI3P at the membrane, it is likely that the function of GL50803_16653 is related to sorting to the PVs. However, no indication of PI3P enrichment has been described so far in the PVs in *G. lamblia*, making the function of GL50803_16653 uncertain. 

Epsin N-terminal homology (ENTH) domain: The ENTH is an evolutionarily conserved domain described primarily in the ENTH/ANTH/VHS protein superfamily that participates in clathrin-mediated endocytosis. The ENTH domain defines monomeric adaptor proteins described as epsin or epsin-related (epsinR), which have the ability to interact with PI4,5P2 at the plasma membrane or with PI4P at the Golgi membrane, respectively, in mammalian and yeast cells [16,17]. Recently, we described the function of the monomeric adaptor protein in *G. lamblia* (GL50803_3256), termed GlENTHp (for *G. lamblia* ENTH protein), the human influenza hemagglutinin (HA)-tagged native version of which was able to bind to PI3,4,5P3 and PI4P, while the HA-tagged ENTH domain alone bound to PI3,4,5P3 in PLO assays [18,19]. This unique protein fulfils a dual function, as a classical epsin protein in receptor-mediated endocytosis (RME) via the tetrameric clathrin-adaptor AP-2, clathrin, and ubiquitin, and like epsinR as a critical factor in ER-to-PVs trafficking of proteins, acting in concert with AP-1 adaptors [18]. 

The PX domain: Proteins containing the phagocyte NADPH oxidase phox-(PX) domain have been described in many cell types and have been implicated in vesicular trafficking, cell signaling, and protein sorting, among other functions [20,21]. Like the FYVE, the PX domains interact with PI3P but may also bind to other PIs [22,23]. They have a preference for the endosomal compartments, although they can also be found in other membranes [24]. In *G. lamblia*, six different PX domains containing proteins have been described on the basis of an in silico searching of the GiardiaDB (http://giardiadb.org/giardiadb/), Assemblage A isolate WB GL50803) [25]. The PX domains of these proteins (GL50803_16596, GL50803_42357, GL50803_16548, GL50803_24488, GL50803_7723, and GL50803_16595) contain the characteristic canonical PX domains, and all but two include basic amino acids, essential for binding the negatively charged PIP head group. PIP binding of their PX domain, cellular localization, and protein expression were determined for these proteins and are listed in Table 1. Although most of the PX-containing domain proteins possess other additional domains, the in silico analysis of the sequence of these *G. lamblia* proteins did not show other special sequences [25]. However, the partial characterization of these PX domain-only proteins in *G. lamblia* might be associated with their role in protein trafficking. For instance, sorting nexins (SNX), proteins containing a PX-domain that binds to PI3P and other PIPs enriched in endosomal membranes [26], have been suggested as part of the retromer complex [27]. These SNXs contain an additional BAR (Bin, amphiphysin, Rvs) motif, which mediates the recruitment of the retromer complex to the endosomal membrane, driving tubule formation [28]. Nevertheless, the participation of non-BAR SNX3 has also been described as mediating the recruitment of the cargo-selective retromer complex [29]. Two proteins, GL50803_24488 and GL50803_16548, described by Jana et al., 2017 [24], possess high sequence similarity to the non-BAR SNX proteins, SNX15 and SNX17, respectively [30,31,32]. The data obtained so far suggest that GL50803_24488 and GL50803_16548 participate in the recovery of membrane proteins from the PVs, where they have the capacity to bind to PI3P and the cargo subunit of the retromer to drive non-tubular transport to the ER in *G. lamblia* [30,31,32].

Another example of PX domain-containing proteins is the t-SNARE Vam7p, a yeast t-SNARE localized to the vacuole [33]. Vamp7p participates in the docking and fusion of specific vesicles to the lysosomal vacuole, acting as a SNAP-25 homologue [33]. In *G. lamblia*, 12 SNARE-like proteins have been found [34], but none of them contains a PX domain. Since non-SNAP-25 family proteins are present in the *G. lamblia* genome, it is possible that, in contrast to Vamp7p, PI3P-specific PX domains of the SNAREs are not required for appropriate PV protein localization.

Except for GlENTHp, the functional properties of PIP-binding proteins have not been analyzed. In the case of GlENTHp, it was found that a reduction of its expression caused a defect in *G. lamblia* cell growth, probably associated with the alteration of the parasite nutrition and PVs homeostasis [18]. These results and those showing that PIP-associated proteins are related to PV structure and function have expanded the knowledge of the critical role of PVs in *G. lamblia* cell survival and may help in the design of new therapeutic parasiticide molecules. 

## 3. Take Me Home: Integral Protein Targeting

Lysosomes play a central function not only in degrading biomacromolecules but also in nutrient and pathogen sensing and membrane repair [35]. Moreover, it was demonstrated that some secretory cell lysosomes, called secretory lysosomes or lysosome-related organelles (LROs), contain proteins destined for secretion in addition to the lysosomal hydrolases [36]. These dual-function organelles are unusual in that they serve both as a degradative and as a secretory compartment. Similarly, *G. lamblia* possesses PVs that function not only as endo-lysosomal compartments but probably also as sorting stations [37,38]. Besides these particularities, protein sorting and delivery to the PVs seem to follow canonical routes described in other cells. For instance, it is well known that lysosomal integral membrane proteins (e.g., LAMP/LIMP family proteins) are transported from the Golgi apparatus to lysosomes by binding their cytosolic motifs to AP complexes [39]. The μ subunits of AP-1, AP-2, and AP-3 bind directly to YXXØ-type sequences, while the γσ1, ασ2, and δσ3 hemicomplexes bind to the [DE]XXXL[LI] sequences [40,41]. In *G. lamblia*, we reported that a type I-membrane cysteine protease (GL50803_14566), termed ESCP (encystation-specific cysteine protease), is transported to the PVs through the tyrosine-based YRPI motif located at its cytoplasmic tail [42]. The YRPI is essential for AP-1 binding and transport from the ER exit site to the PVs in growing trophozoites [42,43]. Also, similar to the mannose 6-phosphate receptors (MPRs) (and Vps10p in yeast), in which the trafficking of the MPR-hydrolase complex to endosomes depends on sorting signals present in the receptor cytosolic tails [44], Vps receptor (GL50803_28954) delivery to the PVs in *G. lamblia* depends on a YQII (YXXØ-type) motif present in its cytosolic tail [45]. This motif is essential for AP-1 interaction and trafficking of the soluble acid phosphatase to the PVs, where it functions as a degradative enzyme [45]. 

Another known type I-membrane protein that cycles between the PM of trophozoites and the PVs throughout a tyrosine-based motif in *G. lamblia* is GlLRP (*Giardia* Low-density lipoprotein Receptor-related Protein, GL50803_113565) [46]. GlLRP shares the substrate-N-terminal binding domain and a FXNPXY-type endocytic motif with human LRP1 and internalizes both low-density lipoproteins (LDL) and chylomicrons, as shown by in vitro studies [46]. The FXNPXY motif of GlLRP was shown to bind directly to the μ2 subunit of AP-2, this being necessary for its proper localization, processing, and function. 

A quick analysis of the proteins described reveals that the trafficking of integral membrane proteins to specific organelles depends on particular motifs in this parasite. However, this may be true only for integral membrane proteins delivered to the PVs. For example, the mitosomal GiMOMP35 protein contains two transmembrane domains in its N-terminal region and is embedded in the outer mitosomal membrane [47]. The targeting of this membrane protein does not rely on a sorting signal composed of a sequence of amino acids but rather on the presence of both transmembrane domains [47]. 

Similar to GlENTHp, alteration (in the expression, transport, etc.) of any of the integral membrane proteins that travel to the PVs causes a defect either in cell growth or in the differentiation to the resistant cyst form [18,37,38,42,43]. This are further data supporting the usefulness of altering PV function as a way of controlling parasite growth and dissemination.

## 4. The Ultimate Fate: Default Targeting to the Plasma Membrane

The trafficking of integral membrane proteins to the plasma membrane has been extensively studied for the variant-specific surface proteins (VSPs), including the specific domains involved in their transport [48,49]. However, there are other integral proteins, like the type I High Cysteine Non-variant Cyst protein (HCNCp) or the DHHC-CDR proteins, for which, although their localization has been defined in a particular membrane, the transport pathway or the domains involved in their trafficking remain unexplored. 

**Figure 1:**
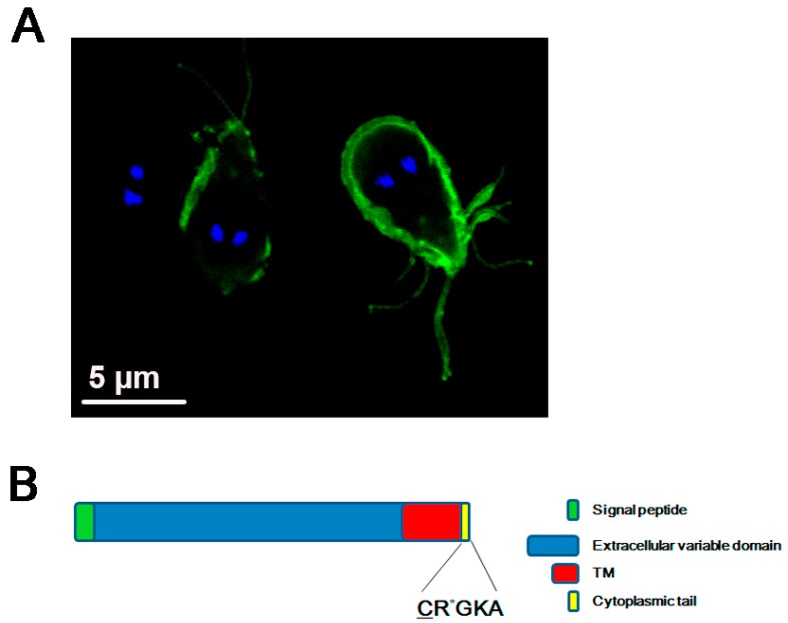
Variant-specific surface protein or VSP. (**A**) Confocal immunofluorescence image showing the plasma membrane localization of the VSP9B10 (green) by using FITC-labeled anti-9B10 mAb. The nuclei are stained with DAPI (blue). Scale bars: 5 μm. VSP9B10 covers two of the trophozoites observed. (**B**) Schematic representation of a VSP containing a signal peptide, an extracellular variable domain, a transmembrane (TM) domain, and the CRGKA invariable cytoplasmic tail. The sites of palmitoylation (underlined C) and citrullination (the asterisk in R) of the cytoplasmic tail are denoted.

The VSPs: Variant-specific surface proteins are a family of proteins that cover the entire surface of the parasite, including the flagella. They are cysteine-rich proteins with particular features: many CXXC motifs, one or two GGCY motifs, zinc finger motifs, a conserved transmembrane region, and an invariable C-terminal tail composed of only five amino acids: CRGKA [50,51,52,53,54]. Each protein contains a highly variable N-terminal region that is exposed to the extracellular media and has been associated with the evasion of host immune response by a mechanism called antigenic variation [55,56,57,58]. The C-terminal regions of these proteins, containing the transmembrane portion and the invariable cytoplasmic tail, have been proposed to be involved in protein trafficking to the parasite membrane [59,60]. In addition, there is evidence that indicates that posttranslational modifications, like protein palmitoylation and citrullination of the C-terminal tail, are directly linked to the process of antigenic variation [48,61] (Figure 1). 

The occurrence of palmitoylation in VSPs was described around 20 years ago and, considering the in vitro experiments indicating that the palmitoylation site was located within the conserved putative transmembrane domain of these proteins, the authors suggested that this fatty acid modification may be involved in anchoring the VSP to the plasma membrane [60,62,63]. This conserved region was suggested as a sorting motif to the plasma membrane. However, some years later, it was demonstrated that neither the amino acid composition of the transmembrane domain nor the C-terminal CRGKA tail were involved in the traffic to the plasma membrane [48]. More recently, an exhaustive study was carried out using mutated VSPs and analyzing their localization, that identified two essential motifs responsible for membrane localization. One of them is located close to the C-terminus and has two CXXCs motifs separated by 12 to 15 amino acid residues, while the other motif is hydrophobic and is also located in the C-terminus [49]. Also, these authors confirmed that neither the C-terminal CRGKA motif nor the N-terminal region are necessary for VSP localization [49].

In contrast with these results, there is another report showing that a chimera, containing the N-terminal leader sequence of a *G. lamblia* cyst wall protein followed by the *Toxoplasma gondii* SAG1 exodomain and the VSPH7 transmembrane domain, is directed to the plasma membrane of trophozoites only if the CRGKA cytoplasmic tail is added [60]. This report, rather than showing the critical role of the CRGKA tail in VSP protein trafficking to the plasma membrane, demonstrates the need to use the correct tools to study how membrane-associated proteins are delivered and integrated into a specific organelle. At least for VSP trafficking, it remains clear that neither the exchange of the transmembrane domain of the VSPs with another sequence similar in length nor the absence of the CRGKA tail alter the fate of VSPs in the plasma membrane of trophozoites, leading to other interesting questions, such as how these two conserved domains participate in the regulation and preservation of antigenic variation in this parasite. For the studies on VSP structure, localization, and function, three VSPs were mainly used: The VSPH7 (GSB_150963) [52], the VSP9B10 or VSP-88 (GL50803_101074) [64], and the VSP/1267 (GL50803_112208) [50]. 

The protein HCNCp (HCNCp, GL50803_40376) was originally described as a VSP-like protein, considering its membrane localization and the presence of multiple CXXC and GGCY motifs. However, it lacks the zinc (Zn) finger motifs present in all VSPs, and both the conserved C-terminal transmembrane region and the short cytoplasmic tail are divergent from those of VSPs [65]. In vegetative growing cells, this protein localizes to the nuclear envelope and the nuclei, but during the process of cell differentiation into cysts, it was found inside the encystation-specific vesicles (ESVs), colocalizing with the cyst proteins during their trafficking to form the cyst wall. At the end of the encystation process, HCNCp localizes to the wall and cell body of cysts [65]. A clear difference between VSPs and HCNCp trafficking is that, while VSPs follow a direct pathway from the ER to the plasma membrane during the entire cell life cycle, HCNCp requires distinct secretory pathways for growth and differentiation, suggesting a significant role of HCNCp in cyst wall formation. So far, the particular function of each region within the HCNCp protein is not known, but it was recently observed that this protein is palmitoylated, although the palmitoylation site and the functional significance of this modification remain unknown [66]. 

The DHHC-CDR proteins: These are a family of polytopic integral membrane proteins containing the conserved Asp-His-His-Cys cysteine-rich (DHHC-CDR) domain required for palmitoyl acyltransferase (PAT) catalytic activity in eukaryotic cells [67,68,69]. The PATs possess three to six transmembrane domains, with the C- and N-terminus facing the cytoplasm. In *G. lamblia*, 9 DHHC-like proteins were identified [66]. The analysis of a group of DHHC proteins showed that three of them localize in the ER and the nuclear envelope, which correlates with the localization observed for HCNCp (nuclear envelope), during growth. Moreover, a partial co-localization of these enzymes with a cyst wall protein suggests that they may be involved in the process of encystation [66]. On the other hand, it was demonstrated that the DHHC GL50803_42184 localizes to the plasma membrane and is involved in the palmitoylation of VSPs [48]. However, there is another DHHC-CDR (GL50803_8711) that localizes to the plasma membrane, but its substrates as well as the domains involved in its localization remain to be elucidated. 

Other integral membrane proteins are described in the GiardiaDB, and some of them have been found by proteomic analysis at different stages of the parasite cell cycle [70,71,72]. These findings and others that allow a more detailed description of the integral membrane proteins in *G. lamblia* will open the doors to a better understanding of these proteins and their role in survival and virulence.

## 5. Conclusions and Future Directions

Complete genomes are available in the GiardiaDB for assemblages A, B, and E, and comparative analyses of these can now be made. Genetic studies and genome sequencing have laid an important foundation for understanding *G. lamblia*. This approach is used to dissect species of the genus *Giardia* and has shown that these assemblages are very diverse, presenting genetic variation between isolates, which appear to possess sufficient differences to be considered distinct [73,74]. Most of the biochemical and cell biological studies have been made using different isolates from the assemblage A (WB1267, C6, etc.), because fusion proteins can be expressed from either episomal or integrated vectors in these trophozoites [75]. There is only one report of integrated transfection of assemblage B (isolate GSH7) [76] and no evidence of transfection of assemblage E exists so far. In Table 2, we depict annotated proteins that show a high degree of sequence similarity between assembles, compared with the ones described in this review. This data might help to perform comparative analyses regarding the differences in infectivity, virulence, and specificity of each assemblage.

**Figure 2:**
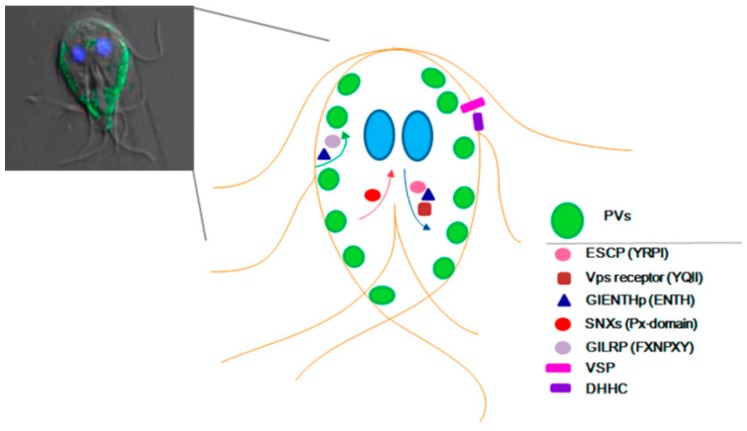
Schematic representation of *Giardia lamblia* proteins localization. Left panel, confocal microscopy merged image showing differential interference contrast, encystation-specific cysteine protease (ESCP)-HA localization in the PVs (green), and the nuclei labelled with DAPI (blue). The right panel shows a draw of proteins that travel from the plasma membrane to the PVs (green arrow), those that use the retromer transport (red arrow), the ones that go from the ER to the PVs (blue arrow), and the integral membrane proteins. Only the proteins with demonstrated experimental localization are denoted. SNXs: sorting nexins.

Compared with what was reported in other organisms, there are limited studies regarding the role of membrane-associated proteins (besides VSPs) in *G. lamblia* (Figure 2). In fact, most of the findings have been collateral to the analysis of diverse mechanisms associated with the parasite’s survival. However, emerging results sustain a critical role of membrane-associated proteins in the physiology of the parasite and disclose the role of proteins and lipids in the control of antigenic variation and cell differentiation into resistant forms. This may lead to the discovery of key molecules or pathways to interrupt the parasites’ infection and reinfection. This relies not only on the similarities found between *G. lamblia* and other cell types but also on the differences. The findings described in this review show the importance of the function of membrane-associated proteins. However, there are still many unanswered questions: how do the expression and membrane association of proteins, like FYVE domain- and PX domain-containing proteins, impact on the origin, maintenance, and function of the PVs? Are lipid modifications critical for HCNCp localization, function, or both? Does the differentiation process change protein localization in trophozoite differentiation into the cyst or vice versa? Answers to these and other emergent questions will certainly help in understanding the peculiarities of *G. lamblia* and in the design of more specific anti-giardial drugs.

## Figures and Tables

**Table 1:** The phagocyte oxidase (PX) domain-containing proteins in *Giardia lamblia*, Assemble A: phosphoinositide-binding specificities, localization and expression. PIP: phosphoinositide; PVs: peripheral vacuoles; ND: not determined; mRNA: messenger RNA.

	GL50803_16596	GL50803_42357	GL50803_16548	GL50803_24488	GL50803_16595	GL50803_7723
**Basic amino acids**	-	-	+	+	+	+
**PX-domain PIP binding (ordered by affinity)**	PI3P	PI3P PI5P PI3,4,5P_3_ PI3,5P_2_ PI4,5P_2_ PI4P	PI3,4P_2_ PI3,5P_2_ PI4,5P_2_ PI3P PI4P PI3P	PI3,4,5P_3_ PI3,4P_2_ PI3,5P_2_ PI4,5P_2_ PI3P PI4P PI3P	PI3,5P_2_ PI3P PI4P PI3P PI4,5P_2_	ND
**Localization in yeast**	Endosomes	Endosomes	ND	ND	Cytosolic vesicles Golgi?	Vacuole membrane
**Localization in *G. lamblia***	ND	ND	PVs	ND	ND	ND
**mRNA expression**	+	+ (mayor expression in cysts)	+	+ (mayor expression in cysts)	+	+

**Table 2:** Orthologs of the membrane-associated proteins of *Giardia* described in the EuPathDB GDB (http://giardiadb.org/giardiadb/). The Gene ID numbers of the proteins described in this work are denoted in light blue. NO: no orthologs. The VSPs are assemblage-specific (*).

	*Giardia* Ortholog Groups				
	assemblage A	assemblage A2	assemblage B		assemblage E
Motiv	Isolate WB	Isolate DH	Isolate GS_B	Isolate GS	Isolate P15
**FYVE**	GL50803_16653	DHA2_16653	GSB_16653	GL50581_4212	GLP15_3543
**ENTH**	GL50803_3256	DHA2_151051	GSB_151711	GL50581_49	GLP15_2242
**PX**	GL50803_16596	DHA2_150243	GSB_150230	NO	GLP15_442
	GL50803_42357	DHA2_150236	GSB_150235	GL50581_3587	GLP15_433
	GL50803_16548	DHA2_16548	GSB_16548	GL50581_1134	GLP15_3433
	GL50803_24488	DHA2_152720	GSB_154119	GL50581_3300	GLP15_1942
	GL50803_7723	DHA2_150244	GSB_150229	GL50581_444	GLP15_443
	GL50803_16595	DHA2_150245	GSB_150228	GL50581_445	GLP15_444
**YXXØ**	GL50803_14566	DHA2_14566	GSB_14566	GL50581_1260	GLP15_3781
	GL50803_28954	DHA2_28954	GSB_28954	GL50581_1526	GLP15_2327
**FXNPXY**	GL50803_113565	DHA2_152495	GSB_153075	NO	GLP15_1378
	GL50803_94510	DHA2_153693			GLP15_1686
**CRGKA (VSP)**	-	-	GSB_150963 *	-	-
	GL50803_101074 *	-	-	-	-
	GL50803_112208 *	-	-	-	-
**CRRSKAV (HCNCp)**	GL50803_40376	DHA2_40376	GSB_40376	GL50581_1041	GLP15_486
**DHHC**	GL50803_42184	DHA2_153342	GSB_154139	GL50581_1824	GLP15_189
	GL50803_8711	DHA2_150040	GSB_150746	GL50581_3650	GLP15_196
